# Daily step count and incident diabetes in community-dwelling 70-year-olds: a prospective cohort study

**DOI:** 10.1186/s12889-020-09929-2

**Published:** 2020-11-30

**Authors:** Marcel Ballin, Peter Nordström, Johan Niklasson, Antti Alamäki, Joan Condell, Salvatore Tedesco, Anna Nordström

**Affiliations:** 1 Department of Community Medicine and Rehabilitation, Unit of Geriatric Medicine, Umeå University, Umeå, Sweden; 2 Department of Public Health and Clinical Medicine, Section of Sustainable Health, Umeå University, Umeå, Sweden; 3 Department of Physiotherapy, Karelia University of Applied Sciences, Joensuu, Finland; 4 School of Computing, Engineering and Intelligent Systems, Ulster University, Londonderry, UK; 5 Tyndall National Institute, University College Cork, Cork, Ireland; 6 School of Sport Sciences, UiT the Arctic University of Norway, Tromsø, Norway

**Keywords:** Physical activity, Walking, Accelerometry, Metabolic syndrome, Non-communicable disease, Visceral adipose tissue, Obesity

## Abstract

**Background:**

Older adults with diabetes take fewer steps per day than those without diabetes. The purpose of the present study was to investigate the association of daily step count with incident diabetes in community-dwelling 70-year-olds.

**Methods:**

This prospective cohort study included *N* = 3055 community-dwelling 70-year-olds (52% women) who participated in a health examination in Umeå, Sweden during 2012–2017, and who were free from diabetes at baseline. Daily step count was measured for 1 week using Actigraph GT3X+ accelerometers. Cases of diabetes were collected from the Swedish National Patient Register. The dose-response association was evaluated graphically using a flexible parametric model, and hazard ratios (HR) with 95% confidence intervals (CI) were calculated using Cox regressions.

**Results:**

During a mean follow-up of 2.6 years, diabetes was diagnosed in 81 participants. There was an inverse nonlinear dose-response association between daily step count and incident diabetes, with a steep decline in risk of diabetes from a higher daily step count until around 6000 steps/day. From there, the risk decreased at a slower rate until it leveled off at around 8000 steps/day. A threshold of 4500 steps/day was found to best distinguish participants with the lowest risk of diabetes, where those taking ≥ 4500 steps/day, had 59% lower risk of diabetes, compared to those taking fewer steps (HR, 0.41, 95% CI, 0.25–0.66). Adjusting for visceral adipose tissue (VAT) attenuated the association (HR, 0.64, 95% CI, 0.38–1.06), which was marginally altered after further adjusting for sedentary time, education and other cardiometabolic risk factors and diseases (HR, 0.58, 95% CI, 0.32–1.05).

**Conclusions:**

A higher daily step count is associated with lower risk of incident diabetes in community-dwelling 70-year-olds. The greatest benefits occur at the lower end of the activity range, and much earlier than 10,000 steps/day. With the limitation of being an observational study, these findings suggest that promoting even a modest increase in daily step count may help to reduce the risk of diabetes in older adults. Because VAT appears to partly mediate the association, lifestyle interventions targeting diabetes should apart from promoting physical activity also aim to prevent and reduce central obesity.

**Supplementary Information:**

The online version contains supplementary material available at 10.1186/s12889-020-09929-2.

## Background

Diabetes increases the risk of retinopathy, nephropathy, physical disability, and cardiovascular disease (CVD), as well as disease-specific and all-cause mortality [1–4]. In addition, diabetes poses a significant economic burden to care systems [5]. Today, the prevalence of diabetes is estimated to exceed 460 million people, [6, 7], and despite some improvements in diabetes-related complications, a considerable burden persists [8]. Meanwhile, the projected rise in diabetes prevalence by 25% until 2030 and 51% by 2045 is largely attributed to rapid population ageing [7, 9]. Thus, effective and sustainable strategies for preventing diabetes among older people should be a priority.

Older people experience increased risk of diabetes due to complex interactions between genetics, lifestyle and biological ageing [10]. The ageing process has a negative effect on β-cell function and leads to increased visceral adipose tissue (VAT) and loss of muscle mass, which together with a decline in physical activity (PA) promotes inflammation and insulin resistance [10, 11]. Lifestyle interventions, focusing on diet and PA, are key components in the management and prevention of diabetes [12, 13], and its effectiveness among middle-aged individuals has been shown in randomized trials [14, 15]. However, research on older people is needed [16] in order to develop appropriate and effective strategies which could help healthcare systems to successfully meet the challenges that will follow the unprecedented ageing of the world’s population.

Walking is the fundamental unit of locomotion and the most readily accessible type of PA and exercise, with a high preference among older people [17]. Self-reported walking has previously been associated with lower risk of incident diabetes [18]. However, the use of wearable devices which can objectively track daily step count is increasing [19], also in older adults [20]. This trend, together with the fact that daily step count is an intuitive metric of PA and something that the general older individual can easily comprehend, implies that findings from such research may have high public health relevance and translational potential. This could further support the development of recommendations and policies [21]. In a recent cross-sectional study, it was found that 70-year-olds with prevalent diabetes take significantly fewer daily steps than those free from diabetes [22]. Against this background, the aim of the present study was to investigate the association of daily step count with incident diabetes in a large cohort of community-dwelling 70-year-old men and women.

## Methods

### Study design and population

This prospective cohort study was based on the Healthy Ageing Initiative study (HAI), initiated in May 2012 in Umeå, a municipality in Northern Sweden with 128,901 inhabitants in 2019. HAI is an ongoing primary prevention study conducted at a single clinic with the aim of identifying traditional and potentially new risk factors for CVD, falls, and fractures among 70-year-olds in Umeå. The eligibility criteria are residence in Umeå municipality and an age of exactly 70. There are no exclusion criteria, and population registers are used for recruitment. From the total number of 70-year-olds living in Umeå, around 70% have chosen to participate in the study since it was initiated in May 2012 [23]. For the present analysis, all HAI participants during May 2012–October 2017 with available PA measurements and who were free from diabetes at baseline were included. The HAI study and the present analysis were both approved by the Regional Ethical Review Board in Umeå, Sweden (no. 07-031 M with extensions), and all participants provided written consent to participate in the study.

### Baseline assessment

In HAI, the participants arrived at the clinic after having fasted for at least 4 h, before they completed a comprehensive test battery where multiple parameters of health were evaluated. Five trained research nurses collected all data with the support of two chief physicians (AN and PN). Waist- and hip circumference were measured using a measuring tape. Body height and weight were measured using a stadiometer (Holtain Limited, Crymych, Dyfed, UK) and digital scale (Avery Berkel HL 120, Taiwan), whereby the body mass index (BMI, kg/m2) was calculated. Blood pressure was measured using a digital automatic blood pressure monitor; Omron M6 Comfort HEM-7221-E (Omron Healthcare, Kyoto, Japan), after at least 15 min of rest. Fasting blood glucose was measured using the HemoCue 201 RT system (Radiometer Medical ApS, Denmark). Blood lipids were measured venously, and the samples were analyzed at the accredited laboratory at the department of clinical chemistry, Umeå University hospital. VAT was measured using a Lunar iDXA device with the CoreScan application (GE Healthcare Lunar, Madison, WI, USA). Participants reported smoking status and prevalent diabetes. Finally, all participants were sent home for 1 week with an accelerometer to assess PA.

### Exposure assessment

Daily step count was measured for 1 week using hip-mounted Actigraph GT3X+ accelerometers (Actigraph, Pensacola, FL, USA). The participants were instructed to be normally active during these days and wear the accelerometer at all times except for when sleeping, showering or bathing. The raw accelerometer data were collected at 30 Hz and filtered using the standard Actigraph filter to eliminate non-human accelerations. Using Actilife software 6.11.3, the raw data were transformed into counts of movement in 60 s epoch lengths, with the activity threshold set to > 100 cpm (CPM), and sedentary time classified as < 100 CPM. Participants were required to have accumulated ≥10 h wear time/day for ≥4 days to be included in the analysis, where non-wear time was identified as periods of ≥ 60 consecutive minutes of 0 counts, with an allowance of maximum 2 min of 0–100 CPM. Step count was determined as per the manufacturer’s proprietary algorithm and was based on vertical accelerations. To calculate mean daily step count (steps/day), the total number of steps during the registration period was divided by the number of valid days that each participant had been wearing the accelerometer. Daily step count measured over the course of 7 days using the Actigraph GT3X+ has previously shown to correlate well to PA energy expenditure as measured by doubly layered water [24].

### Assessment of other variables

Data on diagnoses were collected from the Swedish National Patient Register which covers all inpatient care in Sweden since 1987 and all specialist outpatient care since 2001. Data on prescribed drugs were obtained from the Prescribed Drug Register, which covers all prescribed drugs sold at pharmacies in Sweden since July 2005. To identify deaths during follow-up, we obtained data on all-cause mortality from the Swedish Cause of Death Register. These three registers are managed by the Swedish National Board of Health and Welfare. Socioeconomic data (education, income and marital status) were collected from Statistics Sweden.

### Ascertainment of diabetes

The outcome of the present study was incident cases of diabetes until December 31, 2017. Cases of diabetes were collected from the National Patient Register using the International Classification of Diseases, 10th ed. diagnostic codes E10 and E11. Diagnosis of diabetes in the National Patient Register has a positive predictive value of 79–100% [25].

### Data linkage

We sent the HAI data, along with each participant’s unique personal identity number (issued to all residents of Sweden upon birth or immigration) to Statistics Sweden. Statistics Sweden attached the socioeconomic data and replaced the unique personal identity numbers with a unique study identifier and sent these unique study identifiers to the Swedish National Board of Health and Welfare, who attached the data on diagnoses, drugs and mortality.

### Statistical analysis

To evaluate the association between mean steps/day (continuous variable) and incident diabetes, we calculated hazard ratios (HR) and 95% confidence intervals (CI) using Cox proportional hazard regression models. An evaluation of the scaled Schoenfeld’s residuals showed that the proportional hazards assumption was not violated. Follow-up time was calculated as the number of days from participation in HAI until diagnosis of diabetes, or until death. For participants who were neither diagnosed with diabetes nor died, follow-up time ended on 31st of December 2017. To test for nonlinearity, a squared term for the exposure was added to the model. When the model indicated a nonlinear association (*P* < 0.05), the dose-response association was further evaluated using a flexible parametric model with cubic splines in default positions and three degrees of freedom [26]. Based on this graphic illustration, the association of incremental cut-points for daily step count (dichotomous variable; above/below cut-point) with incident diabetes was investigated using Cox models in 500-steps/day increments, in order to determine the cut-point which best distinguished participants with the lowest risk of diabetes during the follow-up. Adjustment for covariates were performed in several steps. The first model was adjusted only for sex and accelerometer wear time. The second model was additionally adjusted for VAT to investigate its potential role as a mediator in the association between daily step count and diabetes. The third model was additionally adjusted for sedentary time, level of education (primary, secondary, post-secondary), and a composite variable including other cardiometabolic risk factors and diseases. This composite variable was defined as the number of the following risk factors present: anticoagulants; elevated triglycerides (≥ 1.7 mmol/l or lipid-lowering agents); elevated blood pressure (≥ 130/85 mmHg or antihypertensives); and previous stroke/myocardial infarction/angina pectoris. To determine possible effect modification by sex, an interaction analysis was performed by creating a product term between sex and daily step count, which was added to a Cox model adjusted for all covariates listed above.

Next, after we had determined the cut-point at which the association between daily step count and diabetes was strongest, we compared the risk of incident diabetes for participants below this cut-point to participants in categories of 1000-steps/day increments in Cox models according to the 1st and 2nd level of adjustment, as described above.

In a supplementary analysis, we also investigated the association between daily step count and diabetes when adjusting for BMI vs VAT, and how each of these were associated with diabetes when entered simultaneously to the model.

Finally, we conducted a sensitivity analysis to minimize the potential risk of reverse-causality bias. This bias could occur due to early cases of diabetes and short follow-up, and from the fact that a low daily step count at baseline in some participants may be due to early onset, or previous history of disease, as opposed to volitional inactivity. Therefore, we repeated all Cox models after excluding participants with a follow-up time of < 6 months and participants with history of stroke or myocardial infarction. All analyses were performed using Stata version 13.0 (StataCorp, College Station, TX, USA) and SPSS version 25.0 (IBM Corporation, Armonk, NY, USA). *P*-values < 0.05 and CIs for the HRs which did not overlap 1.0 were considered to be statistically significant.

## Results

### Participant characteristics

During May 2012–October 2017, there were 3618 participants in HAI, of which 3291 were free from diabetes. Of these, an additional 236 were excluded due to ineligible accelerometer data. Thus, the total study cohort comprised 3055, 70-year-olds (52%, women), with a mean BMI of 26.2 kg/m2 and a mean fasting blood glucose was 5.5 mmol/l (Table 1). Fewer than 5% had previously suffered a stroke or myocardial infarction, while 54 and 38% had been prescribed anti-hypertensive medication and lipid-lowering agents, respectively. Mean steps/day was 7139 (median 7455).

**Table 1:** Participant characteristics of the study cohort comprising 3055, 70-year-old men and women who participated in the Healthy Ageing Initiative during May 2012–October 2017

**Age, years**	70.5 (0.1)
**Female sex, n (%)**	1589 (52.0)
**Currently smoking, n (%)**	119 (3.9)
**Body mass index, kg/m**^**2**^	26.2 (4.0)
**Waist circumference, cm**	93 (12)
**Visceral adipose tissue, grams**	1423 (921)
**Systolic blood pressure, mmHg**	139 (17)
**Diastolic blood pressure, mmHg**	82 (9)
**Low-density lipoprotein cholesterol, mmol/l**	3.4 (1.0)
**High-density lipoprotein cholesterol, mmol/l**	1.6 (0.5)
**Total cholesterol, mmol/l**	5.6 (1.2)
**Triglycerides, mmol/l**	1.3 (0.6)
**Fasting blood glucose, mmol/l**	5.5 (0.8)
**Steps/day**	7193 (3072)
**Sedentary time, hours/day**	8.8 (1.3)
**Annual disposable income at age 60, 1000 Swedish kronor**	246 (176)
Missing data, n	0
**Education,**^**a**^ **n (%)**	
Primary	524 (17.2)
Secondary	1231 (40.3)
Post-secondary	1296 (42.5)
Missing data, n	4
**Marital status,**^**a**^ **n (%)**	
Married	2036 (66.6)
Never married	253 (8.3)
Widowed	240 (7.9)
Divorced	526 (17.2)
Missing data, n	0
**Diagnoses, n (%)**	
Stroke	98 (3.2)
Myocardial infarction	119 (3.9)
Angina pectoris	205 (6.7)
Fracture	487 (15.9)
Rheumatoid arthritis	65 (2.1)
Renal failure	23 (0.8)
Chronic obstructive pulmonary disease	50 (1.6)
Parkinson’s disease	21 (0.7)
Depression	601 (19.7)
Alcohol intoxication	26 (0.9)
Cancer	562 (18.4)
**Prescribed drugs**^**b**^**, n (%)**	
Antihypertensives	1649 (54.0)
Lipid-lowering agents	1160 (38.0)
Anticoagulants	1091 (35.7)
Hypnotics	805 (26.4)
Sedatives	314 (10.3)
Immunosuppressants	91 (3.0)

Data are presented as means (standard deviation) unless stated otherwise

^a^Education and marital status recorded in the calendar year before the baseline date

^b^Prescribed drugs filled since July 2005

### Risk of diabetes during follow-up

**Fig. 1:**
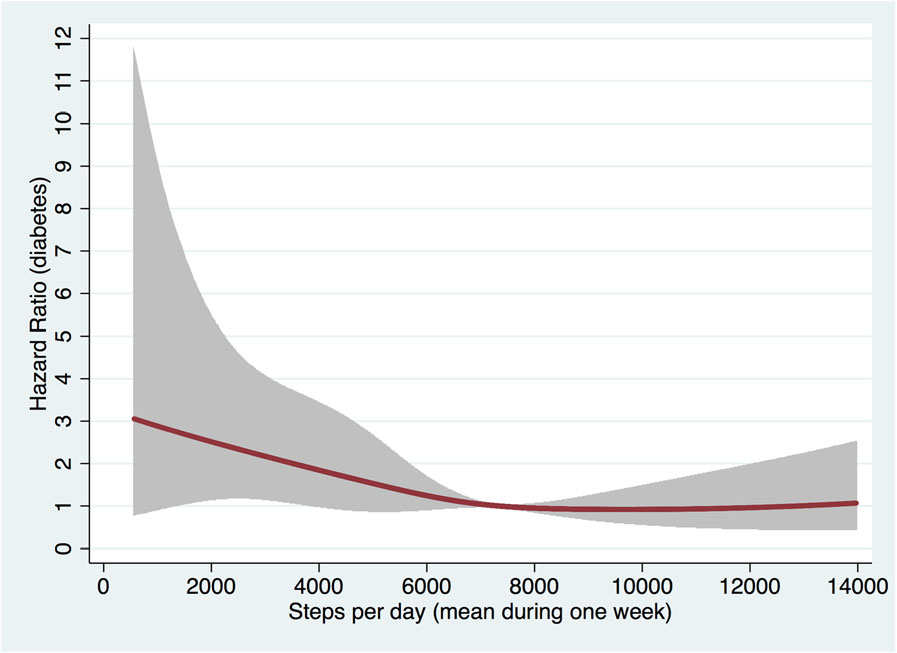
Dose-response association between mean daily step count and incident diabetes. The shaded area represents 95% confidence intervals using a reference of 7445 steps/day which was the median amount of steps/day in the cohort

The mean follow-up time was 2.6 (range 0.1–5.6) years. Diabetes was diagnosed in 81 individuals (26 in women vs 55 in men), 50 individuals died, and the remaining 2924 individuals were followed until 31st December 2017. The incidence rate (IR) of diabetes was 10.2 per 1000-person-years. Mean steps/day was not associated with the risk of incident diabetes (*P* = 0.11) after adjusting for the influence of sex and accelerometer wear time, however, addition of the quadratic term for steps/day to the model suggested that the association was nonlinear (*P*_nonlinearity_ = 0.01). Subsequently, the dose-response association was evaluated graphically using a flexible parametric model with three degrees of freedom. As shown by the dose-response trajectory (Fig. 1), the greatest benefit of a higher daily step count was observed at the lower end of the activity range. From around 6000 steps/day the risk appeared to decrease at a slower rate, before leveling off at around 8000 steps/day. Based on this figure, we investigated which stepping cut-point that best discriminated participants with lower risk of diabetes. Thus, we investigated the association between different cut-points for steps/day with incident diabetes starting at 4000 steps/day up to 6000 steps per day (Table 2). After adjusting for sex and accelerometer wear time, the cut-point at which the association between daily step count and diabetes was strongest was at a cut-point of 4500 steps/day. Specifically, participants taking ≥ 4500 steps/day had 59% lower risk of incident diabetes compared to participants taking fewer steps (HR, 0.41, 95% CI, 0.25–0.66). Further adjustment for VAT attenuated the association (HR, 0.64, 95% CI, 0.38–1.06), and the association was similar in model 3 after further adjustment for sedentary time, education and other cardiometabolic risk factors and diseases (HR, 0.58, 95% CI, 0.32–1.05). Other significant covariates in model 3 included VAT (HR, 1.77, 95% CI, 1.45–2.16 per 1 standard deviation [SD, 921 g] greater VAT) and other cardiometabolic risk factors and diseases (HR, 1.30, 95% CI, 1.06–1.59 per additional risk factor present). The association was not different in men and women *P*_interaction_ = 0.8).

**Table 2:** Hazard ratios for incident diabetes in the study cohort according to different cut-points for daily step count

Stepping cut-points	No. of participants	IR	Hazard ratio (95% CI)		
			Model 1^**a**^	Model 2^**b**^	Model 3^**c**^
**≥ 4000 steps/d**	2707	9.0			
**vs**			0.48 (0.27–0.83)	0.81 (0.45–1.45)	0.79 (0.41–1.53)
**< 4000 steps/d**	348	19.6			
**≥4500 steps/d**	2557	8.2			
**vs**			0.41 (0.25–0.66)	0.64 (0.38–1.06)	0.58 (0.32–1.05)
**< 4500 steps/d**	498	21.0			
**≥ 5000 steps/d**	2399	8.3			
**vs**			0.50 (0.31–0.80)	0.77 (0.47–1.27)	0.75 (0.43–1.32)
**< 5000 steps/d**	656	17.4			
**≥ 5500 steps/d**	2240	8.2			
**vs**			0.55 (0.35–0.88)	0.85 (0.52–1.38)	0.84 (0.48–1.45)
**< 5500 steps/d**	815	15.6			
**≥ 6000 steps/d**	2040	8.3			
**vs**			0.62 (0.39–0.96)	0.95 (0.59–1.54)	0.93 (0.54–1.60)
**< 6000 steps/d**	1014	14.1			

*Abbreviations*: *CI* Confidence interval, *IR* Incidence rate per 1000 person-years

^a^adjusted for sex and accelerometer wear time

^b^additionally adjusted for visceral adipose tissue

^c^additionally adjusted for sedentary time, level of education, elevated triglycerides, elevated blood pressure, antihypertensives, lipid-lowering agents, anticoagulants, myocardial infarction, stroke and angina pectoris

**Fig. 2:**
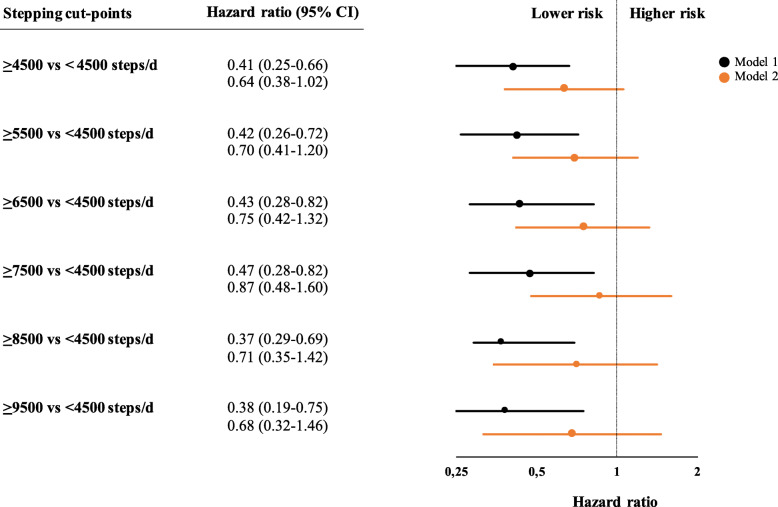
Association between different cut-points of daily step count and incident diabetes. Hazard ratios and 95% confidence intervals are derived from Cox regressions. Model 1 was adjusted for sex and accelerometer wear time and model 2 was additionally adjusted for visceral adipose tissue. Abbreviations: CI, confidence interval

Next, we investigated the association between daily step count and diabetes by comparing participants in categories of 1000-steps/day increments compared to participants below the cut-point of 4500 steps/day (Fig. 2). These analyses suggested a similar pattern, that is, the most pronounced benefit of a higher daily step count occurs early on the spectrum. Specifically, the associations for higher cut-points ranging from 5500 steps/day until 9500 steps/day were similar to the association for the cut-point of 4500 steps/day (Fig. 2). Again, adjusting for VAT attenuated the associations.

The supplementary analysis showed that adjusting either for BMI or VAT resulted in similar associations between daily step count and diabetes (Additional file 1). However, when entering both variables simultaneously to the model, BMI was no longer associated with diabetes (HR, 1.06, 95% CI, 0.98–1.14) whereas VAT was strongly and independently associated with increased risk of diabetes (HR per SD VAT, 1.57, 95% CI, 1.19–2.07).

### Sensitivity analysis

After excluding early cases of diabetes, participants with a follow-up time < 6 months, and participants who had previously suffered a stroke or myocardial infarction, a total of 2715 participants and 58 cases of diabetes were included in the sensitivity analysis. The results of this analysis confirmed the results from the main analysis. Specifically, there was a significant association between the cut-point 4500 steps/day and incident diabetes after adjusting for sex and accelerometer wear time (HR, 0.53, 95% CI, 0.29–0.96), while further adjusting for VAT attenuated the association (HR, 0.75, 95% CI, 0.40–1.40). In model 3, the association was slightly stronger and remained non-significant (HR, 0.63, 95% CI, 0.31–1.29).

## Discussion

In this prospective cohort study, we observed an inverse nonlinear dose-response association between daily step count and incident diabetes in community-dwelling 70-year-olds. There was initially a steep decline in relative risk of diabetes with more steps per day, until around 6000 steps/day from where the risk decreased at a slower rate, before it leveled off at around 8000 steps/day. It was also found that a threshold of 4500 steps/day was best at discriminating participants with lower risk of diabetes, where 70-year-olds who took ≥ 4500 steps/day had around 60% lower risk of incident diabetes compared to those taking fewer steps. However, the association was attenuated after adjusting for VAT.

Today, a lay belief is that one needs to take 10,000 steps every day in order to stay healthy, despite that the scientific evidence to support this statement is weak. On the contrary, a compelling body of evidence shows that compared to being inactive, performing any amount of PA is associated with health benefits, as shown in meta-analyses for the outcome of diabetes, cardiovascular mortality and all-cause mortality [27–29]. Interestingly, although self-reported walking has previously been associated with lower risk of diabetes [18], a recent meta-analysis was unable to definitely characterize the association between daily step count and dysglycemia, partly due to the limited number of studies on this topic [29]. This conclusion confirmed what was highlighted in the 2018 report by the US Physical Activity Guidelines Advisory Committee, which urged researchers to investigate the link between step volume and health outcomes [22]. Thus, the results of the present study provide important knowledge and evidence to the field by showing that a higher daily step count is associated with lower risk of incident diabetes in older adults. In particular, our findings highlight a distinct benefit of daily step counts far below 10,000 steps/day, as our results suggest that the greatest benefits occur when moving away from being inactive to increasing daily activity by even a slight amount. This is supported by the results from two previous prospective studies which observed that even small increments in steps per day were indeed significantly associated with lower risk of incident diabetes in individuals with impaired glucose tolerance [30], and with incident dysglycemia in individuals with normal glucose tolerance [31]. Another important finding of the present study was that the association of daily step count with incident diabetes was independent of sedentary time. Because older adults are sedentary for a majority of their awake time [32], the present findings may have momentous value for sedentary older adults in particular, as to them, an initial goal of at least 4500 steps/day is likely far more attainable than 10,000/day.

As mentioned previously, a threshold of 4500 steps/day was best at discriminating participants with lower risk of diabetes in the present cohort. Participants who took ≥ 4500 steps/day had around 60% lower risk of incident diabetes compared to those who took fewer steps, which was attenuated to around 40% after adjusting for VAT, as well as additional covariates. When we further investigated the risk of diabetes for participants in higher categories of steps/day, from 5500 until 9500, compared to those below 4500, the results were similar. Our study may have had limited possibility to detect other significant thresholds due to insufficient statistical power, as the number of cases was small, still our findings are similar to those observed in a recent study with a larger sample. Lee et al. studied the association of step volume with all-cause mortality in 16,741 older women, with 504 events of death during 4.3 years of follow-up [33]. They found that a cut-point of 4400 steps/day was associated with the largest risk reduction, and from there, the risk decreased at a slower rate until it leveled off at around 7500 steps/day. This pattern is strikingly similar to the one observed in the present study. Together, the encouraging findings from the present study, supported by the previous research, indicates that a daily step count far below 10,000 steps/day may have substantial benefits related to diabetes risk in older adults.

The mechanisms by which PA could influence the risk of diabetes are numerous. For example, PA increases oxidative capacity and promotes insulin-sensitivity and insulin-independent uptake of blood glucose to the working musculature by translocation of glucose transporters, namely GLUT4, to the cell membrane [34–36]. There are also mediating pathways, such as through fat loss. Obesity strongly increases the risk of diabetes in older people [37], and in the present study we found that VAT was a strong risk factor for diabetes in older adults, and partly mediated the association between daily step count and diabetes. This extends the findings from a previous study which found that fat loss mediated the association of daily step count with insulin-sensitivity [38]. There are several potential explanations to the strong link between VAT and cardiometabolic abnormalities [11]. For example, VAT is characterized by high lipolytic activity and has a pro-inflammatory profile, as expressed by its high infiltration of macrophages and the production and secretion of pro-inflammatory cytokines [39, 40]. Given its location, VAT is drained through the portal vein, which may increase the deposition of free fatty acids and pro-inflammatory cytokines in the liver, eventually contributing to hyperglycemia, insulin resistance, and dyslipidemia [39, 40]. In addition, greater VAT is associated with lower levels of adiponectin, which has beneficial effects on insulin sensitivity [39, 40]. Interestingly, we also found that VAT remained strongly associated with diabetes independent of BMI and daily step count. This extends the findings from previous studies showing that VAT is associated with impaired glucose tolerance and diabetes independent of BMI [41–43]. Because ageing is linked to an increase in VAT [44], interventions aiming to mitigate the rising diabetes prevalence in older people may need to be tailored carefully to effectively promote daily PA at a sustainable level, while at the same time reduce VAT.

The findings from the present study may have important implications for clinical practice with respect to the rise in wearable technology, and that the use of devices that can track daily steps is increasing also in older people [20]. This would not only apply to large-scale PA surveillance, but also for wide distribution of PA interventions, as wearable devices have the potential to facilitate behavior change [45]. Based on several meta-analyses, short-term interventions using step counters have been shown to increase daily steps by 2000–2500 steps/day, with favorable effects also on body weight and blood pressure [46–48]. With respect to the outcome of diabetes, it is interesting that a previous study which provided 3 months of light-intensity walking of around 5500 steps/day, improved insulin-resistance and reduced VAT [49]. There are also indications that significant changes in daily step count from short-term interventions may persist in both adults and older adults even 4 years after the intervention [50]. Assuming that additional studies investigate the association between daily steps and health outcomes, with subsequent pooling of their results, the use of wearable devices for tracking daily steps and providing step promoting interventions may eventually become an important and integrated working method in medical care. Especially in terms of management and prevention of non-communicable disease such as diabetes.

### Limitations and strengths

There are several methodological aspects of this study that should be addressed. First, the observational design does not allow causal inferences to be made, although randomized trials have previously proven the effectiveness of lifestyle interventions on diabetes [14, 15]. The relatively short follow-up time in the present study may have increased the risk of bias due to reverse causation, however the associations were confirmed in a sensitivity analysis excluding early cases and participants with short follow-up as well as previous CVD. Still, residual confounding may remain as it has previously been suggested that at least 5 years of follow-up need to be excluded to minimize reverse-causality bias [51]. Next, although the Swedish National Patient Register includes all cases of diabetes from inpatient- and secondary outpatient care, it does not include primary care data, meaning that the incidence of diabetes in the present study may have been underestimated. The few cases also prohibited us from performing extensive subgroup analyses. For instance, stratification based on sex and VAT would have been interesting as we previously observed that the higher prevalence of diabetes in older men compared to women was related to the larger amount of VAT that older men carry [42]. It is also worth mentioning that while similar studies often analyze the associations in multiple categories, this was not deemed suitable in the present study given the few cases as this would compromise the statistical power of our analyses. It would also limit the possibility to include the same number of covariates, which would be a clear disadvantage given that we had access to a vast number of objectively measured parameters including data from national registers with high precision. Finally, it should be mentioned that the Actigraph GT3X+ may result in a slight underestimation of step count, but still correlates strongly with other commercial activity devices on the market [52].

The present study also has several strengths including its prospective design, the large population-based sample with no exclusion criteria, and the inclusion of several important covariates, all of which were objectively assessed. For example, VAT was measured using DXA, and education, prescribed drugs and medical history were all obtained from national registers. Another strength is the objective assessment of daily step count, which not only has high translational potential, but is also a more accurate measure in contrast to self-reported PA, as the latter may lead to biased estimations. For example, in a previous study based on the same population who constituted the present cohort, self-reported PA was more than twice that of objectively measured PA [22], and a recent meta-analysis found the association of objective PA with mortality to be twice as strong as what has previously been found in studies using self-reported measures [27]. Together, the above-mentioned factors increase both the internal and external validity of our findings.

## Conclusions

In summary, this study shows that a higher daily step count is associated with lower risk of incident diabetes in community-dwelling 70-year-olds in a nonlinear dose-response fashion.

The greatest benefits occur at the lower end of the activity range, and far below 10,000 steps/day, with a cut-point of 4500 steps/day found to best discriminate participants with a lower risk of diabetes. The association is independent of sex, education, sedentary time, and cardiometabolic risk factors and diseases, although VAT appears to partly mediate the association. With the limitation of being an observational study, these findings suggest that the greatest benefit of increasing physical activity in relation to the risk of diabetes in older adults is observed when moving from being inactive to being slightly active, and that even a modest increase in daily physical activity is beneficial. Because the association was independent of sedentary time, the results may have implications for sedentary older adults in particular. Finally, as VAT was a mediator in the association, lifestyle interventions aiming to prevent diabetes in older adults should apart from promoting physical activity also aim to prevent and reduce central obesity.

## Supplementary Information

**Additional file 1.** Hazard ratios for incident diabetes in the study cohort according to different stepping cut-points, body mass index and visceral adipose tissue.

## Data Availability

The data analyzed in the present study was a combination of data obtained through clinical examinations in the HAI study and data obtained from national registers. Registry data included socioeconomic data from Statistics Sweden, data on diagnoses from inpatient- and secondary outpatient care from The Swedish National Patient Register, data on prescribed drugs from the Swedish Prescribed Drug Register, and mortality data from the Swedish Cause of Death Register. The individual level data will not be publicly available in accordance with the General Data Protection Regulation. A decoded dataset including the data analyzed during the current study may however be available from the corresponding author on reasonable request.

## Abbreviations

BMI: Body mass index
CI: Confidence interval
CVD: Cardiovascular disease
HAI: Healthy Ageing Initiative
HR: Hazard ratio
PA: Physical activity
SD: Standard deviation
VAT: Visceral adipose tissue
