# Supplementary material for: Daily step count and incident diabetes in community-dwelling 70-year-olds: a prospective cohort study
Source: BMC Public Health. 2020 Nov 30;20:1830. doi: 10.1186/s12889-020-09929-2 (PMC7706282; doi:10.1186/s12889-020-09929-2)
Supplement: Supplementary file 1 — Additional file 1. Hazard ratios for incident diabetes in the study cohort according to different stepping cut-points, body mass index and visceral adipose tissue. [file 12889_2020_9929_MOESM1_ESM.docx]

| **Supplementary Table.** Hazard ratios for incident diabetes in the study cohort according to different cut-points for daily step count, body mass index and visceral adipose tissue. | | | | |
| --- | --- | --- | --- | --- |
|  | **Hazard ratio (95% CI)** | | | |
| **Stepping cut-points** | **Model 1^a^** | **Model 1 + BMI** | **Model 1 + VAT^a^** | **Model 1 + BMI and VAT^a^** |
|  |  |  |  |  |
| **>4000 vs <4000 steps/d** | 0.48 (0.27-0.83) | 0.80 (0.44-1.44) | 0.81 (0.45-1.45) | 0.87 (0.48-1.58) |
| BMI (kg/m^2^) |  | 1.15 (1.10-1.21) | - | 1.07 (0.99-1.15) |
| VAT (per SD greater) |  | - | 1.87 (1.56-2.26) | 1.59 (1.21-2.09) |
|  |  |  |  |  |
|  |  |  |  |  |
| **>4500 vs <4500 steps/d** | 0.41 (0.25-0.66) | 0.63 (0.37-1.06) | 0.64 (0.38-1.06) | 0.68 (0.40-1.15) |
| BMI (kg/m^2^) |  | 1.14 (1.09-1.20) | - | 1.06 (0.98-1.14) |
| VAT (per SD greater) |  | - | 1.81 (1.51-2.18) | 1.57 (1.19-2.07) |
|  |  |  |  |  |
|  |  |  |  |  |
| **>5000 vs <5000 steps/d** | 0.50 (0.31-0.80) | 0.77 (0.46-1.28) | 0.77 (0.47-1.27) | 0.83 (0.50-1.38) |
| BMI (kg/m^2^) |  | 1.15 (1.10-1.21) | - | 1.06 (0.99-1.15) |
| VAT (per SD greater) |  | - | 1.86 (1.54-2.23) | 1.59 (1.20-2.09) |
|  |  |  |  |  |
|  |  |  |  |  |
| **>5500 vs <5500 steps/d** | 0.55 (0.35-0.88) | 0.84 (0.51-1.37) | 0.85 (0.52-1.38) | 0.91 (0.56-1.50) |
| BMI (kg/m^2^) |  | 1.15 (1.10-1.21) | - | 1.07 (0.99-1.15) |
| VAT (per SD greater) |  | - | 1.88 (1.56-2.26) | 1.59 (1.21-2.10) |
|  |  |  |  |  |
|  |  |  |  |  |
| **>6000 vs <6000 steps/d** | 0.62 (0.39-0.96) | 0.90 (0.56-1.46) | 0.95 (0.59-1.54) | 1.01 (0.62-1.65) |
| BMI (kg/m^2^) |  | 1.16 (1.10-1.21) | - | 1.07 (0.99-1.15) |
| VAT (per SD greater) |  | - | 1.90 (1.58-2.30) | 1.60 (1.21-2.11) |
|  |  |  |  |  |

Model 1 was adjusted for sex and accelerometer wear time
^a^ 1 SD VAT = 921g

**Abbreviations:** BMI = body mass index, CI = confidence interval, SD = standard deviation, VAT = visceral adipose tissue
